# A comparative look at structural variation among RC–LH1 ‘Core’ complexes present in anoxygenic phototrophic bacteria

**DOI:** 10.1007/s11120-020-00758-3

**Published:** 2020-05-19

**Authors:** Alastair T. Gardiner, Tu C. Nguyen-Phan, Richard J. Cogdell

**Affiliations:** 1 Institute of Molecular, Cellular and Systems Biology, College of Medical, Veterinary and Life Sciences, University of Glasgow, Glasgow, G12 8QQ UK; 2 Laboratory of Anoxygenic Phototrophs, Centre Algatech, Institute of Microbiology of the Czech Academy of Sciences, Novohradska 237, 379 01 Třeboň, Czech Republic

**Keywords:** Purple photosynthetic bacteria, Light harvesting, Structures, Reaction centres, Anoxygenic phototrophs, RC–LH1

## Abstract

All purple photosynthetic bacteria contain RC–LH1 ‘Core’ complexes. The structure of this complex from *Rhodobacter sphaeroides*, *Rhodopseudomonas palustris* and *Thermochromatium tepidum* has been solved using X-ray crystallography. Recently, the application of single particle cryo-EM has revolutionised structural biology and the structure of the RC–LH1 ‘Core’ complex from *Blastochloris viridis* has been solved using this technique, as well as the complex from the non-purple Chloroflexi species, *Roseiflexus castenholzii*. It is apparent that these structures are variations on a theme, although with a greater degree of structural diversity within them than previously thought. Furthermore, it has recently been discovered that the only phototrophic representative from the phylum Gemmatimonadetes, *Gemmatimonas phototrophica*, also contains a RC–LH1 ‘Core’ complex. At present only a low-resolution EM-projection map exists but this shows that the *Gemmatimonas phototrophica* complex contains a double LH1 ring. This short review compares these different structures and looks at the functional significance of these variations from two main standpoints: energy transfer and quinone exchange.

## Introduction

The intra-cytoplasmic membranes (ICM) of anaerobic phototrophic bacteria contain all the pigment-protein complexes necessary for photosynthetic growth. In the ICM, the light-harvesting (LH) antenna that is intimately associated in a complex with a type-2 reaction centre (RC) is known as the RC–LH1 ‘Core’ complex and forms the ‘heart’ of the photosynthetic unit (PSU). In many species, the PSU consists of both a RC–LH1 ‘Core’ complex and peripheral antenna complexes termed LH2. However, there are species of anoxygenic phototrophic bacteria, e.g. *Rhodospirillum *(*Rsp.*)* rubrum* and *Blastochloris *(*Blc.*)* viridis* that contain PSUs with only RC–LH1 ‘Core’ complexes (i.e. no LH2) and are perfectly able to sustain photosynthetic growth (Aagaard and Sistrom 1972; Eimhjellen et al. 1963).

To help understand fully the function of the ICM, it is necessary to have high-resolution structures of the constituent protein complexes. The development and availability of a broad range of mild, non-ionic and zwitterionic detergents has had a dramatic effect upon the study of membrane biology and the proteins present in photosynthetic membranes are no exception. The go-to approach, at least until recently, was to obtain high-resolution structural information by growing protein crystals of the desired complex for X-ray crystallography (these complexes are generally not yet suitable for NMR analyses (Liang and Tamm 2016; McDermott 2009).The ability to easily and reproducibly purify stable, intact complexes to homogeneity has led to structural determination of the RC and LH2. However, for many years equivalent structural data about RC–LH1 ‘Core’ complexes were lacking. These complexes have some intrinsic characteristics that mean they are not ideally suited for growing the large, highly ordered crystals necessary for X-ray studies. Compared with most other membrane proteins, there are almost no extra-membrane hydrophilic domains/regions and, the fixed Cytochrome *c* subunit excepted (if present), all the RC–LH1 ‘Core’ complex functionality takes place within the hydrophobic, membrane spanning region. After solubilisation, a large proportion of the complex is, by necessity, shrouded within the detergent micelle and this hinders the formation of strong, stable, protein–protein crystal contacts. The fixed Cytochrome c and the cytoplasmic-side H-subunit domain may be able to form good contacts, as they are generally out of the detergent micelle. Such contacts, however, can lead to the fixed Cytochrome c making contact with the H-subunit of a neighbouring molecule in the lattice in a repeating head-to-toe arrangement, resulting in a crystal that is a flat, two-dimensional plate and produces only anisotropic diffraction. Three-dimensional crystals of antenna complexes also have a relatively high solvent content, ~ 70% (Roszak et al. 2003), resulting in the requirement for large crystals that contain a sufficient number of molecules to enable diffracted X-rays to be detected. RC–LH1 ‘Core’ complex crystals that are suitable for X-ray crystallography have, therefore, to overcome the contradictory requirements for large crystals but without the ability to form strong, stable crystal contacts. As the crystal grows, the inherent, long-range disorder in the lattice increases and so it diffracts poorly. Nevertheless, persistent and methodical optimisation of crystals has resulted in RC–LH1 ‘Core’ complex structures using X-ray crystallography from *Rhodopseudomonas *(*Rps.*)* palustris* at 4.8 Å, pdb 1PYH (Roszak et al. 2003) and *Tch. tepidum* at 3.0 Å, pdb 3WMM (Niwa et al. 2014), subsequently improved to 1.9 Å, pdb 5Y5S (Yu et al. 2018).

Over the past few years, structural biology has been revolutionised by the rapid and on-going advancement in cryo-EM technology. Cryo-EM has made possible near atomic level structure determination from single particles quickly, without the need for crystallisation, large amounts of protein or even for the protein to be 100% homogeneous. These advances were recognised by the award of the 2017 Nobel Prize in Chemistry to Jacques Dubochet, Joachim Frank, and Richard Henderson (Nogales 2018).

It is worthwhile to make a brief comparison between the determination of protein structures by X-Ray crystallography and cryo-EM. In principle, given equivalent resolution the two techniques should produce equally reliable structures. However, it is worth examining this a bit more critically. When X-Ray crystallography is used to solve a protein structure, then it is assumed that all the protein molecules in the crystal are identical. In a general sense this is true (otherwise there would not be a crystal), but the technique is not able to differentiate between individual protein molecules in the lattice. A difference electron density map is generated that undergoes an iterative process of refinement using the co-ordinates and structure factors to generate the final, robust structure. Solving a protein structure with cryo-EM involves a more linear workflow but initially makes the same assumption that all the molecules selected from the EM grid are identical. One can select, sort and overlay with the software different populations of individual particles to produce a coulomb potential map of the molecule. Inherent heterogeneity within the particles means that it is more difficult to align all the regions molecules equally and results in a distribution of resolution across the map. It is important to bear in mind, therefore, that the meaning of ‘resolution’ is slightly different for a cryo-EM structure compared with an X-ray structure. The ability to computationally select particles means that it is possible to look for changes in protein structure between populations. In this way, cryo-EM can detect conformational forms of proteins that would be hard, if not impossible, to visualise using X-Ray crystallography.

Within a very short timescale, research into photosynthetic complexes has benefitted greatly from the application of cryo-EM: for complexes that previously produced only poorly diffracting crystals (or were not able to be crystallised at all) and to multi-complex super assemblies that would be extremely difficult to crystallise intact, for example the phycobilisome, with cryo-EM structures already available from the red alga *Griffithsia pacifica* at 3.5 Å, pdb 5Y6P (Zhang et al. 2017) and *Porphyridium purpureum* at 2.82 Å, pdb 6KGX (Ma et al. 2020). The application of cryo-EM has now produced high-resolution structures for the RC–LH1 ‘Core’ complex from *Blc. viridis* to 2.9 Å, pdb 6ET5 (Qian et al. 2018) and the RC–LH *Roseiflexus *(*Rof.*)* castenholzii* to 4.1 Å, pdb 5YQ7 (Xin et al. 2018).

With the availability of these RC–LH1 ‘Core’ complexes, obtained using both X-ray crystallography and cryo-EM, it has become clear that there is a much greater degree of structural variation within them than previously considered (Cogdell and Roszak 2014). In this concise mini-review, the structures are presented in a historical context and compared by highlighting the respective similarities and differences. It is neither our intention nor wish to reproduce the fine structural details (residues, distances, H-bonds, etc.) that are characteristic of each complex. Interested readers should refer to the corresponding publications, where these features are covered fully. Table 1 provides an overview of the main features from the different structures, which are then described in more detail below.

**Table 1:** A comparison of the RC–LH1 ‘Core’ complexes from the anoxygenic phototrophs mentioned in the text

Species	Resolution	Method	LH1 ring shape	LH1 apoproteins	LH1 pigments	RC pigments	RC subunits	Quinone/quinol exchange	Other subunits
*Rps. palustris*	4.8 Å	X-ray	Elliptical ring with gap	15 αβ	30 Bchl a (B880)	Not resolved	L, M, H	Protein W-induced channel	Protein W
*Rba. sphaeroides*	8 Å	X-ray/NMR/EM/MS	LH1 ribbon enclosing two RC	28 αβ	56 Bchl a (B875): 56 sphaeroidene	2 x (4 Bchl *a*:4 Bphe\*a*) 2 × sphaeroidene UQ_10_, UQ_10_	L, M, H	PufX-induced channel	Protein PufX
*Blc. viridis*	2.9 Å	Cryo-EM	Elliptical ring with a pore	16 αβγ trimers, 1 αβ dimer	34 Bchl *b* (B1015): 17 1,2-dihydro-neurosporene and dihydro-lycopene	4 Bchl *b*: 2 Bphe *b*: 1,2-dihydro-neurosporene MQ_9_, UQ_9_	L, M, H, C	LH1 ring pore due to the ‘missing’ 17^th^ γ-polypeptide	None
*Tch. tepidum*	1.9 Å	X-ray	Completely closed elliptical ring	16 αβ	32 Bchl a 16 spirilloxanthin	4 Bchl a: 2 Bphe *a*: Spirilloxanthin MQ_8_, UQ_8_	L, M, H, C	No gap/pore, exchange occurs through the LH1 alpha helices	None
*Rof. castenholzii*	4.1 Å	Cryo-EM	Elliptical ring	15 αβ	45 Bchl a (30 B880 15 B800) 14 keto-α-carotene	3 Bchl a: 3 Bphe a No resolved carotenoid MQ_11_, MQ_11_	L, M, C	Channel composed of a fixed Cyt *c* helix, the 15^th^ LHαβ and the flexible transmembrane helix of the subunit X	Subunit X

The table is compiled from the following: *Rps. palustris* (Roszak et al. 2003), *Rba. sphaeroides* (Qian et al. 2013), *Blc. viridis* (Qian et al. 2018), *Tch. tepidum* (Yu et al. 2018), *Rof. castenholzii* (Xin et al. 2018)

In general, all RC–LH1 ‘Core’ complexes must fulfil two basic functions (Barz et al. 1995a, b; Cogdell et al. 2004; McGlynn et al. 1994; Sundström and van Grondelle 1995). Firstly, they provide an excitation energy transfer conduit that links light absorption by the antenna system to charge separation in the reaction centre (RC). Once excited, the RC catalyses the primary, transmembrane electron-transfer reactions that result in a quinone molecule being reduced to a quinol. Secondly, the quinol must then be able to pass through the LH1 ring and equilibrate with the bulk quinone pool in the membrane to maintain the cyclic electron transport pathway via the cytochrome *b/c*_*1*_ complex. This ultimately creates a transmembrane electrochemical proton gradient and drives the synthesis of ATP (Hu et al. 1998; Moser et al. 2003). Studies of RC–LH1 ‘Core’ complexes should always consider these two basic functions, namely energy transfer and quinone/quinol exchange, how they are achieved and the advantages and disadvantages that any particular structural variant confers.

## Discussion

In 1995, an 8.5 Å electron microscopy projection structure of the *Rsp. rubrum* LH1 complex was determined (Karrasch et al. 1995). The 2D crystals in this study were produced from reconstituted, detergent-solubilised αβ-subunits and the projection map revealed a closed, circular 16-mer ring formed from these repeating dimers. This finding was somewhat controversial at the time, as it was thought that a closed ring would render quinone/quinol exchange through the palisade of α-helices impossible. Albeit many years later, the authors were vindicated with the publication of the *Tch. tepidum* RC–LH1 ‘Core’ (Niwa et al. 2014), which also has a closed ring. Subsequently, Jamieson et al. (2002) also published an 8.5 Å projection structure of RC–LH1 ‘Core’ complex from *Rsp. Rubrum*; however, the 2D crystals in this study came from intact, purified complexes rather than reconstituted subunits. Maps were calculated from two different crystal forms, one of which showed a circular LH1 ring and the other an elliptical LH1 ring. This was the first direct, experimental evidence that LH1 rings could be non-circular. Interestingly, later atomic force microscopy (AFM) studies on ICM from *Blc. viridis* revealed that when the LH1 ring is enclosed by a RC, the ring shape is then elliptical. When the RC was nano-dissected out with the AFM tip to leave only the LH1 ring, then it relaxed into a more circular structure (Scheuring et al. 2003). This is strong, direct experimental evidence that the shape of the LH1 ring is modulated by interactions with the RC. AFM has proven to be a very useful technique to show the overall size and shape of the 'RC–LH1 ‘Core' complexes in intact ICM and how they may be organised relative to each other and to LH2 complexes. As such, AFM is an important tool to aid understanding of these high-resolution structures in the context of the native PSU. However, due to its relatively low resolution compared with either X-Ray crystallography or cryo-EM, AFM should be used rather conservatively when trying to determine subunit stoichiometry or gaps within these antenna 'rings'.

**Fig. 1:**
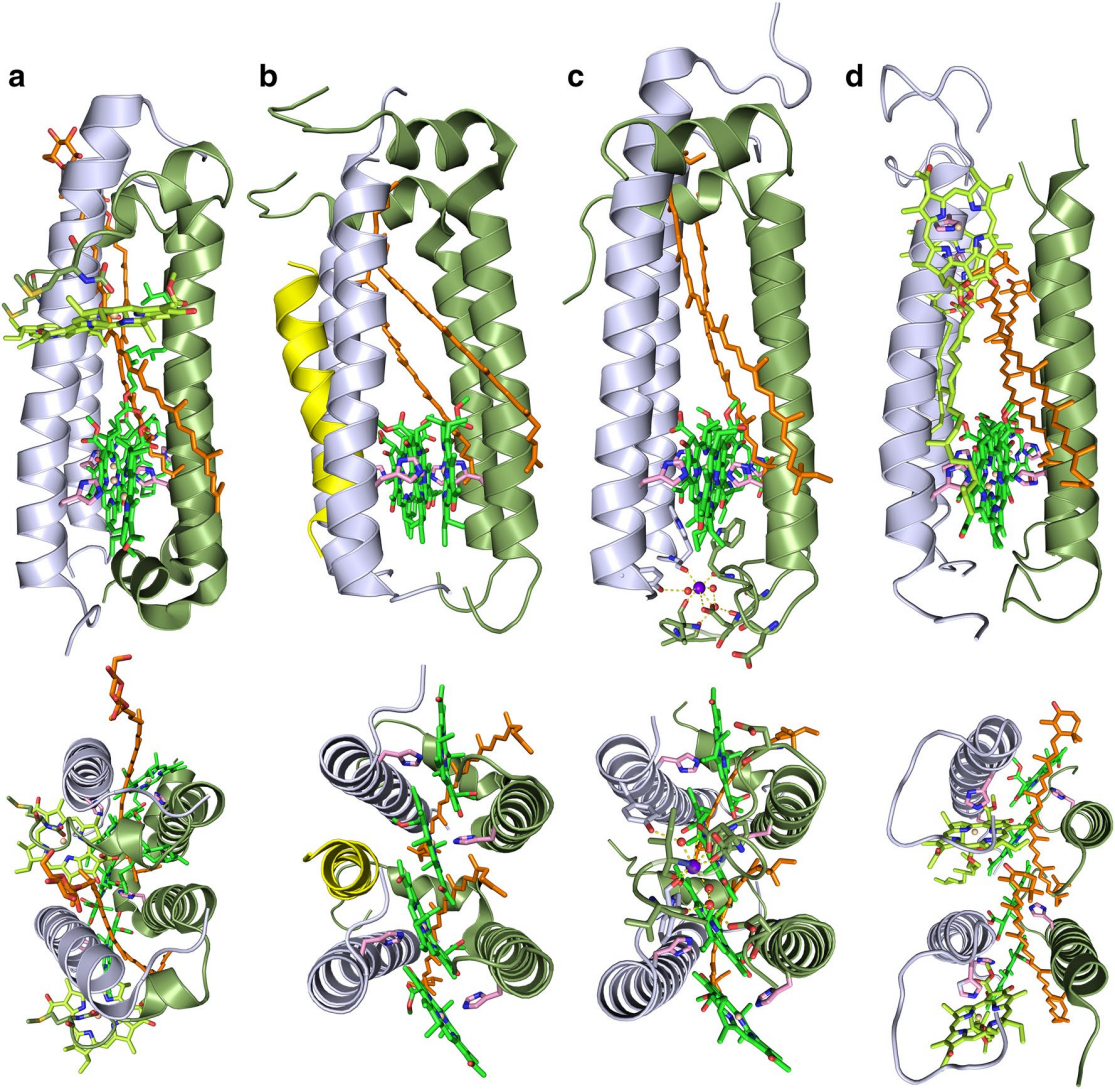
Two αβ-dimers in the ring of the light-harvesting complexes. Each view is slightly adjusted for maximal visibility. The Bchl tails and last few residues from some of the polypeptide chains have been omitted for clarity. The top row is in the plane of the membrane and bottom row is normal to the membrane. Common colours; α-polypeptides—purple blue, β-polypeptides—olive, ring Bchl—green, monomeric Bchl—lemon, carotenoids—orange, coordinating His residues—pink. **a** For comparison, a double αβ-dimer from the LH2 complex from *Rbl. acidophilus*. The monomeric Bchl is liganded to the C-terminal of the α-chain through a carboxy modified α-Met. The lower view is from the periplasmic side. **b** *Blc. viridis* with the additional γ-helix in yellow on the outside of the LH1 ring and between two β-helices. The lower view is from the cytoplasmic side. **c** *Tch. tepidum* with the co-ordinated Ca^2+^ on the cytoplasmic side shown in magenta and the water molecules as red spheres. This network of bonds requires residues from both αβ-dimers. The lower view is from the cytoplasmic side and part of the C-terminal α-polypeptide chain has been made partially transparent for clarity. **d** *Rof. castenholzii* with the monomeric Bchl liganded to a His residue on the α-polypeptide. The lower view is from the cytoplasmic side

Long before there were 3D structures of RC–LH1 ‘Core’ complexes, the primary structure of many LH1 apoproteins was determined by both protein and gene sequencing (Zuber and Cogdell 1995). Alignment of these sequences revealed that they were all highly homologous (Brunisholz and Zuber 1988), relatively short polypeptides of typically 50–60 amino acids (5–7 kDa) and predicted to have hydrophilic ends separated by a single transmembrane spanning α-helix containing a single conserved His residue. This is nicely illustrated in Qian et al. (2018) (Extended data Fig. 9), as these authors published an alignment of some representative LH1 α- and β-polypeptide sequences. CLUSTAL O v.1.2.4 analyses of these sequences reveals that the α- and β-sequences matched against each other have a residue identity of 39% and 43%, respectively. If strongly conserved and/or weakly conserved changes are also taken into account, then that value increases even more. It was suggested that this His residue is co-ordinated to the central Mg atom in the middle of the bacteriochlorin ring of bacteriochlorophyll (Bchl) (Zuber 1987). These main conclusions, all drawn from a rather simple comparison of LH1 apoprotein primary structures, are clearly borne out in all the following structures. The LH1 ‘ring’ that is formed around the RC is made from a repeating oligomer of a basic heterodimeric structure composed of an α- and β-apoprotein pair (Qian et al. 2013; Roszak et al. 2003; Yu et al. 2018), in an analogous manner to LH2 complexes (Gabrielsen et al. 2009), with the light absorbing pigments, Bchl and carotenoids, bound non-covalently. Figure 1 illustrates two of these αβ-dimers together (i.e. as they are in the ring), for the species presented in this review and for which there are high-resolution structures available, along with an equivalent view of a ‘double αβ-dimer’ from the LH2 complex of *Rhodoblastus *(*Rbl.*)* acidophilus* (formerly *Rps. acidophila*) strain 10050 for comparison, pdb 1NKZ. The importance of the conserved His residue (coloured pink in Fig. 1) that co-ordinates to the central Mg atom in the middle of the bacteriochlorin ring is clearly evident. The composition of the αβ-dimer subunit that makes up LH2 complexes is rather standardised, Fig. 1a, consisting of the αβ-dimer, three Bchl *a* and one carotenoid. Depending on the species, these αβ-dimer units can oligomerise to form LH2 complexes with different ring sizes, for example, *Rbl. acidophilus* (McDermott et al. 1995) and *Rps. palustris* (Southall et al. 2018) make a nonameric complex, *Phaeospirillum *(*Phs.*)* molischianum* (Koepke et al. 1996) makes an octameric complex and *Allochromatium vinosum* is thought to make a dodecameric complex (Kereïche et al. 2008). The ring size can change but the composition of the ring remains very similar. However, it is now known that the composition of the subunits that oligomerise to form the LH1 ring in RC–LH1 ‘Core’ complexes is not constant; *Blc. viridis* contains an additional γ-polypeptide (Fig. 1b), *Tch. tepidum* contains a bound Ca^2+^ ion (Fig. 1c) and *Rof. castenholzii* contains an additional Bchl (Fig. 1d). Clearly, LH1 (and LH2) rings are capable of forming many different sizes and it is interesting to ask where and how the information for the oligomeric size is encoded? Perhaps, as it has been suggested (Pugh et al. 1998), the precise way in which the LH1 ring assembles around the RC controls the resultant ring size. Clearly further work is required to answer this fascinating question.

**Fig. 2:**
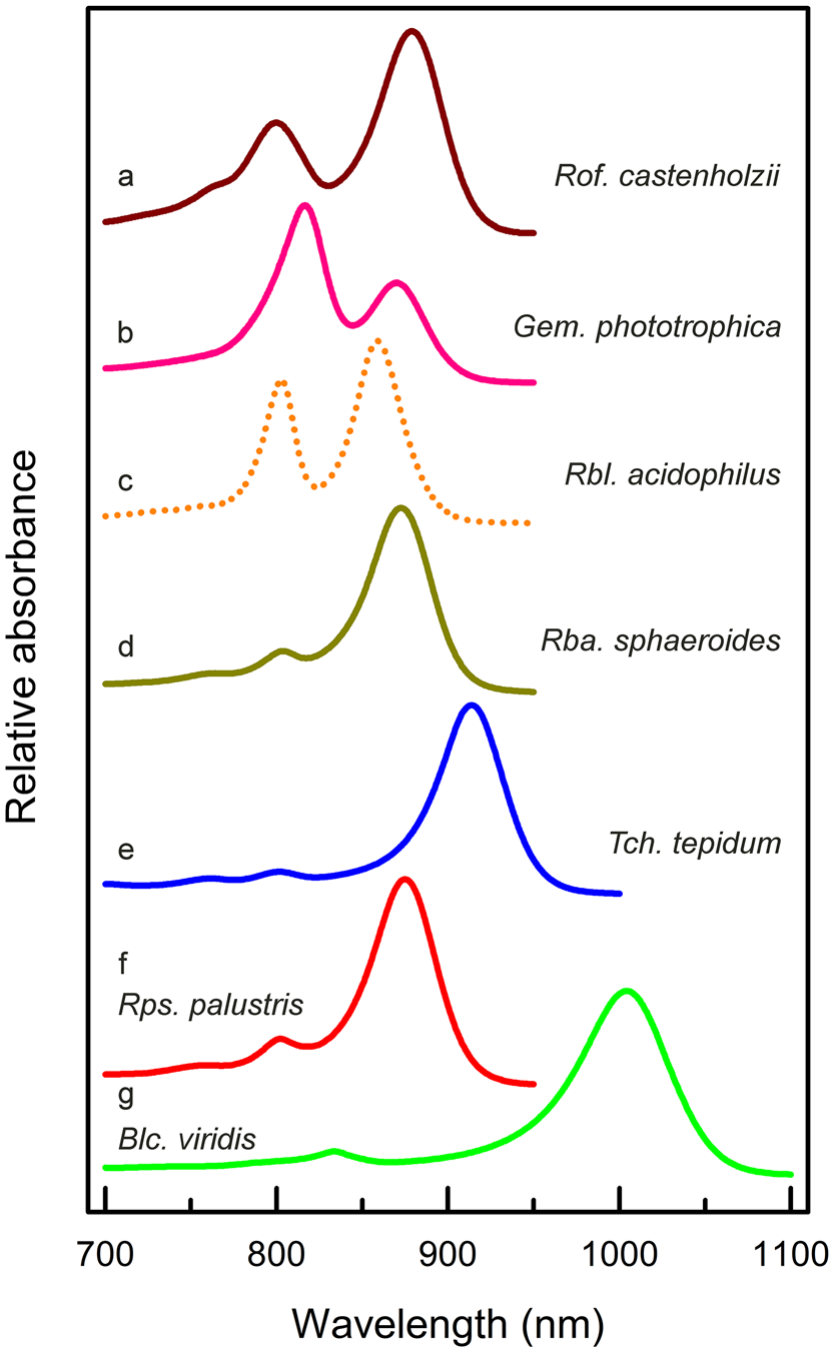
NIR absorption spectra of the RC–LH1 complexes mentioned in the text. The colour assignments are as follows along with the respective complex absorption band maxima (nm); **a** *Rof. castenholzii*, brown (800, 879), **b** *Gem. phototrophica*, pink (816, 868), **c** *Rbl. acidophilus* LH2, orange (804, 858), **d** *Rba. sphaeroides*, olive (873), **e** *Tch. tepidum*, blue (914), *Rps. palustris*, red (875), **d** *Blc. viridis*, green (1004)

The pairs of Bchl are strongly exciton coupled and interact around the LH1 ring to give rise to the well-known single strong Q_y_ absorption band in the near infrared (NIR), illustrated in Fig. 2. It is apparent that some of the absorption spectra are mostly rather similar, differing mainly in the exact position of the peak of the Bchl *a* Q_y_ band. Monomeric Bchl *a* in organic solvents, such as 7:2 (v/v) acetone:methanol, has a Q_y_ band that absorbs at 772 nm (Clayton 1966). The precise reason why these complexes have a different Q_y_ absorption band red shift has been a long-standing open question. The factors that can, in principle, produce red shifts of the Bchl Q_y_ absorption band have been considered in detail elsewhere (Cogdell et al. 2002; Robert et al. 2003). In general, the overall molecular environment of any individual Bchl in its binding pocket sets that Bchl’s site energy. In addition, exciton coupling between the strongly interacting Bchl molecules in the LH1 ring causes a further red shift. The extent of this shift then depends on the precise strength of this exciton coupling as well as other factors such as charge-transfer states (Nottoli et al. 2018). It can be seen from Fig. 2 that *Rof. castenholzii* (Fig. 2a brown) and *Gemmatimonas *(*Gem.*)* phototrophica* (Fig. 1b pink) have RC–LH1 ‘Core’ complex spectra that are markedly different as they have two strong Bchl Q_y_ absorption peaks in the NIR rather than one. The *Rof. castenholzii* complex has a minor band at 800 nm and a major band at 879 nm (Xin et al. 2012; Yamada et al. 2005), whereas the *Gem. phototrophica* RC–LH1 ‘Core’ has a major band at 816 nm and a minor band at 868 nm (Dachev et al. 2017). A typical LH2 complex, such as the one from *Rbl. acidophilus* strain 10050 (Fig. 2c orange dash), also has two NIR Bchl *a* Q_y_ absorption bands, in this case at 804 nm and 858 nm (Gardiner et al. 1993) and is known as a B800-850 complex. There are also other variants of LH2 that have different absorption spectra, for example, *Rbl. acidophilus* strain 7050 makes a ‘standard’ B800-850 complex at high-light but a B800-820 complex (formerly known as LH3) under low-light growth conditions. *Rps palustris* makes a heterologous LH2 complex, in which the B800-850 complex produced under low-light (previously called LH4) has a B850 band absorption that is substantially reduced compared with the high-light adapted ‘standard’ B800-850. In all these LH2 complexes, the population of Bchl *a* molecules that absorb at ~ 800 nm is less strongly excitonically coupled than the population absorbing at (~ 820 to) ~ 850 nm, and so the red shift is not as pronounced. It is apparent, therefore, that the RC–LH1 ‘Core’ complexes from *Rof. castenholzii* (Collins et al. 2010) and *Gem. phototrophica* (Dachev et al. 2017) are different to those previously characterised from purple bacteria. They are RC–LH1 ‘Core’ complexes but also have features of LH2 as they contain two populations of Bchl *a* molecules, one strongly excitonically coupled and the other weakly coupled. The following sections that deal with the *Rof. castenholzii* and *Gem. phototrophica* complexes will reveal the completely different structural solutions adopted by these species, which enable the polypeptides to act as a scaffold for these two (one strongly and one weakly coupled) Bchl *a* populations. For the *Rba. sphaeroides* (Fig. 2d olive), *Tch. tepidum* (Fig. 2e blue) and *Rps. palustris* (Fig. 2f red) spectra, the small peak just to the blue of the main LH1 NIR Bchl *a* Q_y_ absorption band (~ 800 nm) originates from the RC. The RC peak from *Blc. viridis* is at 833 nm (Fig. 2g green).

**Fig. 3:**
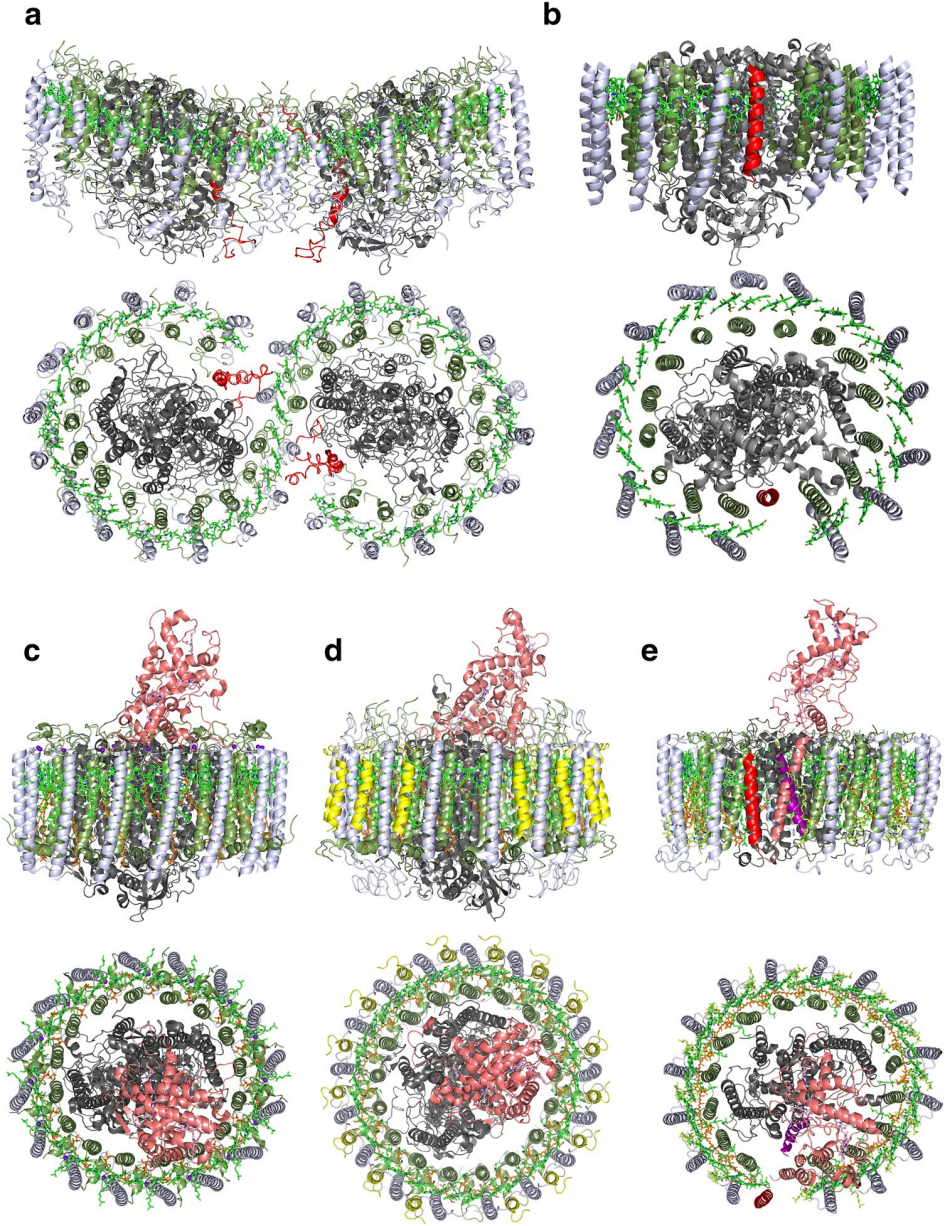
In plane (side on view) and membrane-normal (top side, periplasmic view) views of RC–LH1 complexes. The colour scheme is the same as in Fig. 1 with the fixed Cytochrome *c* given in salmon pink. **a** The dimeric complex from *Rba. sphaeroides* with gap-forming polypeptides PufX protein (red). **b** *Rps. palustris* with the gap-forming Protein W (red). **c** The LH1 ring from the complex in *Tch. tepidum* has no discernible gap and the ring of Ca^2+^ ions (magenta) is visible between each successive αβ-pair. **d** The *Blc. viridis* complex contains an additional smaller γ-polypeptide (yellow) that intercalates between the β-helices on the outside of the ring. The absence of this polypeptide at one site facilitates quinone/quinol exchange. **e** The *Rof. castenholzii* complex with the previously unknown, channel-forming subunit X (red). The channel is also formed by a transmembrane helix that protrudes down from the fixed Cytochrome *c* subunit. The enigmatic RC helix, TM7, mentioned in the text is represented in purple

**Fig. 4:**
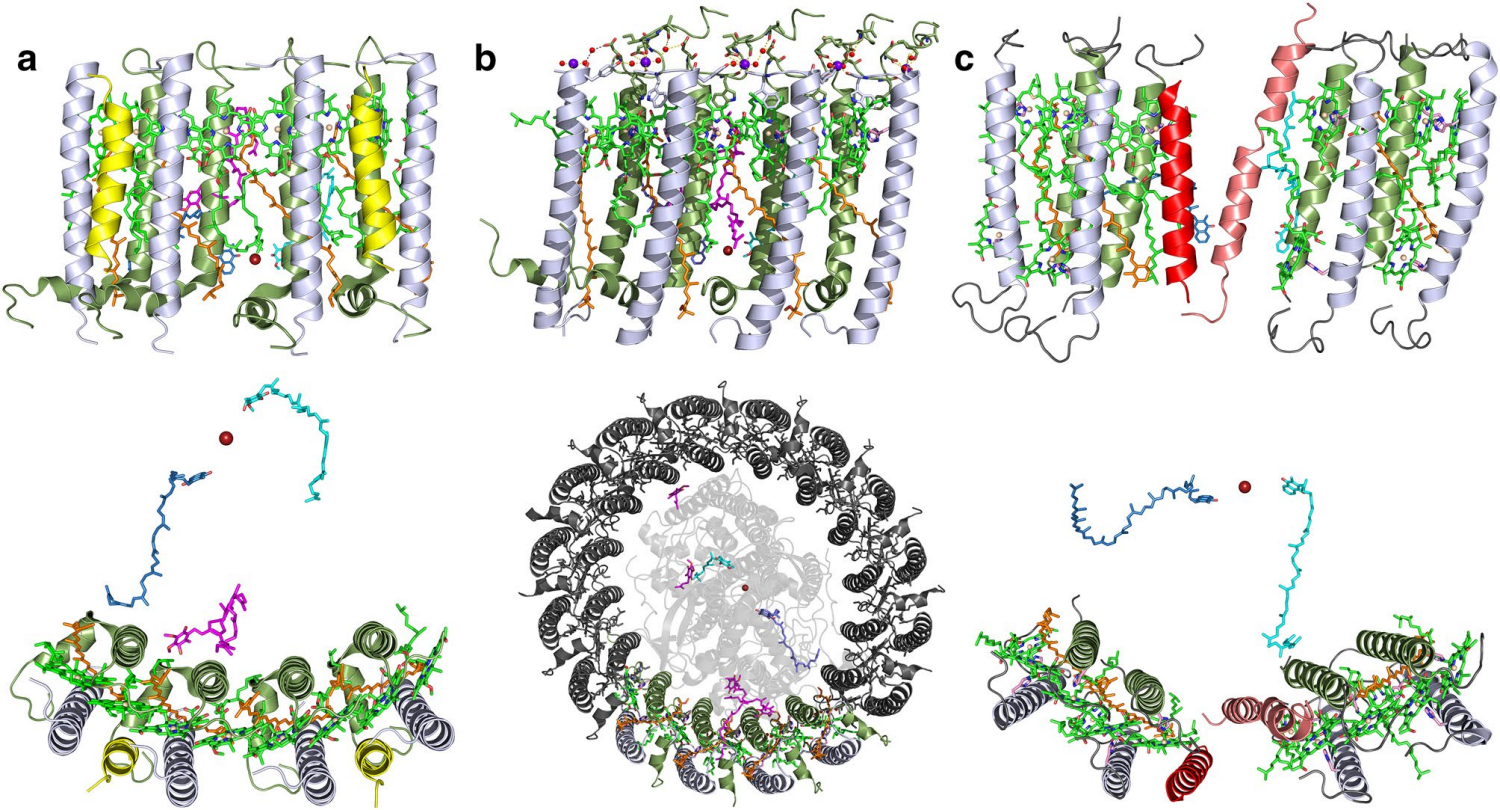
Quinone/quinol exchange mechanisms used in the three high-resolution structures mentioned in the text. The colour scheme is the same as in Fig. 1, with the non-haem iron in firebrick red, Q_A_ quinone in blue, Q_B_ in cyan and any additional quinones resolved in the structures are in magenta. The top row presents a side view of the four αβ-helices in the complex where quinone/quinol exchange is presumed to take place. The bottom row is the equivalent membrane-normal view. Where required, a few of the terminal amino acids have been trimmed from the polypeptides termini in order to aid visibility. **a** *Blc. viridis*, the lower view is from the periplasmic side. **b** *Tch. tepidum*. The lower view shows the complete complex (minus the fixed cytochrome) from the cytoplasmic side with the same ring segment coloured that is presented above. The rest of the ring is given in grey and 80% transparency has been applied to the RC in order to help visualise Q_A_ and Q_B_. **c** *Rof. castenholzii* has a relatively big gap produced by helix X (red) and the helix from the fixed Cytochrome (salmon pink). The lower view is from the periplasmic side

Figure 3 compares a membrane-plane (side) and membrane-normal (periplasmic) view of the overall RC–LH1 ‘Core’ complex structures from; *Rba. sphaeroides*, pdb 4V9G (Fig. 3a) (Qian et al. 2013), *Rps. palustris* (Fig. 3b) (Roszak et al. 2003), *Tch. tepidum* (Fig. 3c) (Yu et al. 2018), *Blc. viridis* (Fig. 3d) (Qian et al. 2018) and the RC–LH complex *Rof. castenholzii* (Fig. 3e) (Xin et al. 2018). Even at first glance, it is straightforward to classify the complexes into two groups, depending on whether they contain one RC with its LH1 ring, or two RCs joined together by a ‘ribbon’ of LH1, i.e., if they are ‘monomers’ or ‘dimers’. Indeed, this classification was used for many years before the details of these newer structures became available. It is now apparent that ‘monomeric’ RC–LH1 ‘Core’ complexes are much more prevalent in Nature than ‘dimeric’ complexes. Outside of the genus *Rhodobacter*, only one other dimeric RC–LH1 ‘Core’ complex has been described, a 13 Å electron microscopy projection structure from *Rhodobaca bogoriensis* (Semchonok et al. 2012). *Rhodobaca* is a haloalkaliphilic genus that is closely related to *Rhodobacter* and together they form the *Rhodobacter-Rhodobaca* (RR) group within the order Rhodobacterales (Kopejtka et al. 2017). It has been determined from extensive 16S rRNA phylogenetic analyses of phototrophic bacteria that only species within the RR group contain the *puf*X gene and, therefore, are able to make ‘dimeric’ complexes. The PufX protein is required for photosynthetic growth and plays a critical role in the dimerisation process as well as quinone/quinol exchange, Fig. 2a (Qian et al. 2017; Tunnicliffe et al. 2006). A more detailed inspection of the structures can also lead to a classification based on the presence or absence of a ‘gap’ in the LH1 ring. This feature is examined in more detail for the different species in Fig. 4. It should be borne in mind, however, that these pictures are drawn expressly to illustrate the way this exchange can occur. Pictures drawn using a (possibly more realistic) space-filling mode would show almost no space in the respective gaps/pores/channels. The importance of the quinone/quinol exchange mechanism had always been appreciated, even if the specific solution adopted by each RC–LH1 ‘Core’ complex had not yet been elucidated. Perhaps, then, the most rewarding finding, for those of us that are interested in these complexes, is the diversity and unexpected structural variety that make each of these complex structures so different and it would not be surprising if even more natural variation remains to be discovered. The individual complexes will now be discussed in more detail.

### The *Blastochloris viridis* RC–LH1 ‘Core’ complex

The ICM from *Blc. viridis* contains large, regular hexagonal arrays of the RC–LH1 ‘Core’ complexes. In the 1980s, these membranes were used in two EM studies that produced low-resolution models of the RC–LH1 ‘Core’ complex (Miller 1982; Stark et al. 1984). The earlier study suggested that these complexes are hexagonal, whereas the second suggested that they are dodecameric. More recently, AFM has been used on these membranes and the results suggested the LH1 component around the RC is hexadecameric (Scheuring et al. 2003). It is, therefore, interesting that the true high-resolution structure has shown that the LH1 component is a 17-mer, Fig. 3d. It is possible that these previous studies all made pre-conceived assumptions about the symmetry of the complex and then processed the data based on those assumptions. The different number of subunits obtained for images of the same complex clearly illustrates the inherent danger if the imposed symmetry is wrong. As previously mentioned, the primary sequences of the LH1 apoproteins were published in the 1980s by the group of Herbert Zuber at ETH Zürich. They found that the RC–LH1 complex from *Blc. viridis* contains α- and β-polypeptides and an additional, smaller non-pigment binding apoprotein, called the γ-subunit, in a 1:1:1 ratio (Brunisholz et al. 1985). Even though this extra, smaller γ-polypeptide was described a long time ago, it was largely forgotten. It is rather rewarding to finally discover, after all this time, how the γ-subunit is organised in the overall structure of the RC–LH1, Figs. 1b and 3d (Qian et al. 2018). The LH1, slightly elliptical ring, is a 17-mer composed of 16 αβγ-trimers and one αβ-pair with no γ-subunit. The γ-subunits occupy a position on the outside of the ring between adjacent β-subunits. The space in the ring where this one γ-subunit is missing enables a ~ 5 × ~ 7 Å pore to be formed and quinone/quinol exchange to take place (Fig. 4a). In the structure, a folded quinone molecule has been visualised out with the Q_B_ site that appears to be in the correct position to pass through the LH1 ring. It may appear from Fig. 4a that the tails of the Bchl and the end of the carotenoid may obstruct this exchange. However, Qian et al. (2018) note that the densities for these parts of the molecules are less strong than in the equivalent molecules elsewhere in the ring and suggest that they have a degree of flexibility so that the pore is not blocked. In all sections of Fig. 4, the quinone occupying the Q_A_ site is illustrated blue, the Q_B_ site quinone in cyan and any additional quinones found are given in magenta.

Unlike the other RC–LH1 ‘Core’ complexes that are described here, the complex from *Blc. viridis* contains Bchl *b* (Eimhjellen et al. 1963), rather than Bchl *a*, and its long wavelength, NIR absorption band, Q_y_, is at 1004 nm (Fig. 2g green). This absorption band is much further to the red than the corresponding band from the other Bchl *a*-containing complexes. A remarkable feature, as the NIR Q_y_ absorption band of monomeric Bchl *b* in organic solvent such as 7:2 (v/v) acetone:methanol is only ~ 15 nm red shifted from that of Bchl *a* in the same solvent (Scheer 1991). As mentioned previously, the overall molecular environment of any individual Bchl in its binding pocket sets that Bchl’s site energy and this is as true for Bchl *b* as it is for Bchl *a*. Parkes-Loach et al. (1990) have shown that reconstitution of the LH1 complex from *Rsp. rubrum* with Bchl *b* resulted in an approximate 30 nm red shift relative to reconstitution with Bchl *a*. Recently, Swainsbury et al. (2019) showed that reconstituting the monomeric Bchl site in the LH2 complex from *Rba. sphaeroides* with Bchl *b* resulted in a 30 nm red shift relative to reconstitution with Bchl *a*. It has been suggested, based on the structure of the *Blc. viridis* RC–LH1 complex, that the additional red shift of the Q*y* absorption band is also due to stronger Bchl: Bchl interactions, as the Bchl *b* molecules are closer together than in the other types of RC–LH1 ‘Core’ complex (Qian et al. 2018). These stronger interactions result in stronger exciton coupling and, therefore, a larger red shift. A full quantum mechanical calculation of the ring is required, using the position of each of the atoms in the structure, to test whether this hypothesis can be validated by theory (such as in Cupellini et al. (2016)). At 2.9 Å, however, the accuracy of any given atom position may not be sufficient, making the calculations unreliable or even not possible. This illustrates two interconnected points, first that small improvements in resolution can and do make real and tangible differences towards understanding the important details of a molecule. Secondly, a full understanding of the function does not come about just by having a high-resolution structure per se, rather this requires further close co-operation between experimentalists and theoreticians and is particularly true for the complexes involved in the light reactions of anoxygenic photosynthesis, as there are no mechanical conformation changes or enzymatic intermediates that can be visualised. The fact that *Blc. viridis* contains Bchl *b* is not just some outlying curio in the world of photosynthetic bacteria. This species is very sensitive to the presence of oxygen (Lang and Oesterhelt 1989) and the resultant red shift of the LH1 NIR Q_y_ absorption band results in a selective advantage for *Blc. viridis* in microaerophilic environments as this enables the cells to absorb solar energy that is unavailable to Bchl *a*-containing anoxygenic phototrophs (Pierson et al. 1990).

### The *Thermochromatium tepidum* ‘Core’ complex

The structure of RC–LH1 ‘Core’ from the thermophilic bacterium *Tch. tepidum* has been determined to 1.9 Å, the highest resolution so far for this type of complex, Fig. 3c (Niwa et al. 2014; Yu et al. 2018). The LH1 component is a 16-mer that forms an LH1 ellipse with no permanent gaps. It is suggested that the molecular motion of the LH1 in the membrane allows the complex to ‘breathe’ and so facilitates quinone/quinol exchange through a channel formed on the interfaces between each pair of adjacent apoprotein dimers (Fig. 4b top). This is supported by the finding in the structure of an isoprenoid tail from one quinone/quinol molecule present between the α- and β-subunits of LH1. The clear implication is that this represents a transient ‘pore’ through which the quinol molecule can pass, even though it is closer to Q_A_ than Q_B_. However, as the αβ-dimer subunits are all similar, the question arises if this is the only ‘pore’ present? One can speculate as to how many such ‘pores’ might exist and to what extent does the presence of the RC influence the ‘breathing’ of the ring (Fig. 4b bottom). These hypotheses need to be tested by detailed molecular dynamic simulations and is another example where theoreticians can make a significant contribution!

As can be seen from Fig. 2e, the *Tch. tepidum* LH1 has its major Bchl *a* NIR Q_y_ absorption band at 914 nm. This red shift is rather less than that for *Blc. viridis* but significantly larger than for the other purple bacterial LH1 NIR Q_y_ Bchl *a* absorption bands, usually found between 875 and 890 nm. This red shift correlates with the presence of tightly bound Ca^2+^ ion, one co-ordinated in the space between each αβ-dimer in the C-terminal loop region, Figs. 1c and 4b top (Kimura et al. 2008, 2009). The ends of the αβ-polypeptides in RC–LH1 (and LH2) complexes are, generally, quite flexible and may not be visible in the electron density maps. However, the presence of Ca^2+^, and the resulting network of bonds, restrains the ends of the polypeptides and has enabled them to be resolved by Yu et al. (2018). These authors successfully managed to determine that the binding site of these Ca^2+^ ions involves, in total, coordination with seven amino acid residues (5 on the α-chain and two on the β-chain) and three water molecules, to form a beautiful bonded ring of Ca^2+^ around the periplasmic side of the complex. Interestingly, other divalent cations can substitute for Ca^2+^ in the *Tch. tepidum* RC–LH1 ‘Core’ complex (Kimura et al. 2018). The structure of this complex has also been solved with either Sr^2+^ or Ba^2+^ substituting for Ca^*2*+^ (Yu et al. 2016). Substitution of the Ca^2+^ with either divalent metal ion results in a complex with the Q_y_ band exhibiting a red shift to only 888 nm. The network of bonds that co-ordinate the Sr^2+^ or Ba^2+^ ions originates only from the α-polypeptide and results in a complex that has much reduced thermostability. Complete removal of these calcium ions also results in a blue shift of the Bchl *a* Q_y_ absorption band. Indeed, when the Ca^2+^-binding motif from *Tch. tepidum* was sequentially engineered into the LH1 antenna polypeptide sequences of *Rba. sphaeroides*, by progressively modifying the native LH1 polypeptides, Ca^2+^ binding was induced, and the extent of the red shift directly correlated with the proportion of *Tch. tepidum* sequence incorporated (Swainsbury et al. 2017). It has been suggested that loss of Ca^2+^ relaxes the structure and weakens the Bchl *a* exciton coupling in the LH1 ring, resulting in a blue-shifted absorption band. It would be useful to have a high-resolution structure for the Ca^2+^-free complex to enable detailed quantum mechanical calculations to probe the possible molecular mechanisms that underlie this blue shift, and quantitatively test how the binding of Ca^2+^ influences the resulting structural rigidity of the complex. Thermostability of the complex induced by Ca^2+^ is important obviously for the organism, *Tch. tepidum* was first isolated from the Mammoth Hot Springs in Yellowstone National Park, but it is also a feature that may be exploited by synthetic biology as a template for engineering increased stability into other proteins.

### The *Rhodopseudomonas palustris* ‘Core’ complex

The structure of the RC–LH1 ‘Core’ complex from *Rps. palustris* was determined by X-ray crystallography to a resolution of 4.8 Å, Fig. 3b (Roszak et al. 2003). At this resolution, the structure should be viewed sensibly, and the limitations conferred at this resolution need to be appreciated. For example, the organisation of the Bchl *a* molecules shown in Fig. 3b looks rather irregular compared with those in the *Tch. tepidum* (Fig. 3c) and results from a lack of detail in the electron density map at this resolution. Single molecule studies on the complex have shown clearly that the actual organisation of these Bchl *a* molecules is indeed more regular (see Fig. 5 in Richter et al. (2007)). The *Rps. palustris* RC–LH1 ‘Core’ complex has an elliptical 15-mer LH1 with a gap in the ring where Protein W (coloured red in Fig. 3b) is located. Protein W appears rather analogous to PufX in the *Rba. sphaeroides* complex, in that it provides the channel through which quinone/quinol exchange can take place. Elegant single molecule spectroscopy experiments have also provided strong evidence for the presence of the gap (Richter et al. 2007). Recently, however, a question has arisen following studies on a RC–LH1 ‘Core’ complex from *Rps. palustris,* where Protein W was His-tagged, whether all the RC–LH1 ‘Core’ complexes from this species actually contain W (Jackson et al. 2018). Further work is needed to clear up this anomaly and this complex is now a prime target for analysis by single particle cryo-EM where the structure should be able to be re-determined rather quickly.

### The *Rhodobacter sphaeroides* ‘Core’ complex

The structure of the RC–LH1 ‘Core’ complex from *Rba. sphaeroides* has been determined by X-ray crystallography to a resolution of ~ 8 Å, with the resultant model built by using constraints provided by EM, NMR and mass spectrometry, Fig. 3a (Qian et al. 2013). It has two RC connected by an ‘S’-shaped ‘ribbon’ of LH1 and so is called a ‘dimeric’ RC–LH1 complex. The dimer is stabilised by the presence of two copies of a protein called PufX (coloured red in Fig. 3a), one for each LH1 ring. Deletion or mutation of PufX, and especially its C-terminus, results in an inability to form the dimer and a monomer is produced (Qian et al. 2017). PufX provides the channel through which the reduced quinones can exit the LH1 rings and connect with the cyclic electron transport pathway. Each LH1 ring consists of one RC, 14 αβ-subunits and one copy of PufX. The role of PufX was first suggested based on a series of papers from the groups of Oesterhelt and Hunter, and then confirmed by constructing deletion strains of *Rba. sphaeroides* (Barz et al. 1995a, b; Barz and Oesterhelt 1994; McGlynn et al. 1994). These groups showed that deletion of the *puf*X gene prevented photosynthetic growth by slowing down the rate of electron transfer from the RC quinol to Cytochrome *b*/*c*_*1*_. This kinetic block could be removed by the deletion of the genes encoding LH1 (Lilburn and Beatty 1992; McGlynn et al. 1994), by point mutations in the LH1 polypeptides that promote a degree of structural rearrangement (Barz and Oesterhelt 1994) or by the removal of a hypothetical second carotenoid binding site (Olsen et al. 2017). The determination of the *Rba. sphaeroides* RC–LH1 ‘Core’ complex structure beautifully provided a structural explanation for these molecular genetic deletion experiments.

### The *Roseiflexus castenholzii* ‘Core’ complex

All the species of anoxygenic phototrophic bacteria mentioned previously belong to the class Alphaproteobacteria, apart from *Tch. tepidum*, which is a Gammaproteobacteria. The filamentous bacteria *Rof. castenholzii* is not a member of the Proteobacteria, rather belongs to the phylum Chloroflexi and was previously classed as a green non-sulphur bacterium. The structure of the RC–LH ‘Core’ complex from *Rof. castenholzii* has been determined by single particle cryo-EM to an overall average resolution of 4.1 Å, Fig. 3e (Xin et al. 2018). Note that these authors prefer to use the term RC–LH rather than RC–LH1 to describe this complex. The structure bears a superficial resemblance to the other ‘monomeric’ complexes presented in Fig. 3 but, perhaps not surprisingly given the evolutionary diversity, has some significant unique differences. The RC is rather different to the RC in proteobacteria; the LM-subunit contains separate L- and M- polypeptides but they are encoded by a single fused *puf*LM gene (Yamada et al. 2005). Six transmembrane helices make up the L subunit and five transmembrane helices comprise the M-subunit. The presumed sixth transmembrane helix of the M-subunit was found to have non-continuous density with the other helices and so was modelled as a separate helix in the structure, termed TM7 (identifier Y in the pdb and coloured purple in Fig. 3e). It is presently unclear whether TM7 really is a separate protein and, if so, whether it is the product of (i) an, as yet, unidentified gene or (ii) results from even more post-translational processing of the nascent PufLM polypeptide than was previously envisioned. The L-M-TM7-subunit contains 3 Bchl *a* and 3 Bacteriopheophytin (Bphe) *a* instead of 4 and 2, respectively, (Collins et al. 2010) and the kinetic properties of the RC are rather similar to other previously studied RC purple bacteria (Collins et al. 2011). There is no indication in these previous studies on the *Rof. castenholzii* RC that any triplet states formed in the Bchl ‘special pair’ P are not being quenched, i.e. the carotenoid in the RC is functioning normally. However, in the structure no RC carotenoid was resolved and the reasons for this are not at all clear. The molecule could well be more flexible than the equivalent in purple bacterial RCs but then one could still expect some of the molecule to be resolved, e.g. the area around the *cis*-bond, but this appears to be not the case. There is one further, particularly intriguing feature of the RC from this species and that is that it contains no H-subunit. This is evident in Fig. 3e and is particularly interesting as it has been previously assumed that the H-subunit plays a central role in the formation of RC–LH1 in the membrane. The H-subunit is inserted first, and this acts as an anchor about which the rest of the RC, and then LH1, is built. As the *Rof. castenholzii* LH–RC has no RC-H, either this assumption is incorrect or the assembly pathway is rather different in Chloroflexi compared with that in all other known anoxygenic phototrophs.

The LH ring contains 15 αβ-subunits but the position where a 16^th^ subunit (e.g. by analogy with the *Tch. tepidum* complex) would be is replaced by a channel formed by a quite novel method. A previously unknown polypeptide, called X (coloured red in Fig. 4c), forms a transmembrane helix and sits on the outer side of the LH1 ring. This is complemented by an extension from the fixed Cytochrome *c* that protrudes downward to form a transmembrane helix on the inner side of the ring (coloured salmon pink in Fig. 4c). These two proteins create the channel through which quinone/quinol exchange is able to take place. The presence in the channel area of transmembrane Helix X and the transmembrane helix from the fixed Cytochrome occupy more space in the structure than just the missing 16th αβ-subunit. This is reflected in the loss of one carotenoid molecule from the ring so that only 14 are present.

The other unique feature of this RC–LH ‘Core’ complex is that the LH αβ-dimer subunit binds three Bchl molecules rather than two, which is typical for the other complexes in Fig. 3, so that there are 45 Bchl *a* molecules per RC (Collins et al. 2010). The αβ-dimer (Fig. 1d) contains the two Bchl *a* molecules that give rise to the ‘normal’ LH1 NIR Q_y_ absorption band at 879 nm and an additional, more weakly coupled monomeric Bchl *a* molecule that produces the second NIR Q_y_ absorption band at 800 nm. Some anoxygenic phototrophic Proteobacteria are able to produce a LH2 complex in the ICM in addition to the RC–LH1, yet other species perform light-harvesting and grow under phototrophic conditions perfectly well without LH2. It is interesting to note the striking similarities between the *Rbl. acidophilus* LH2 αβ-dimer, Fig. 1a, and the *Rof. castenholzii* LH1 αβ-dimer, Fig. 1d, with the only major difference being the different ligation method and orientation of the monomeric Bchl *a*. Usually in photosynthetic complexes, the Mg atom of chlorophyll/Bchl molecules is liganded by histidine residues. In the LH2 complex from *Rbl. acidophilus,* the Mg atom of the monomeric Bchl *a* is ligated via a carboxyl group extension of the N-terminal methionine residue of the α-apoprotein (Fig. 1a) (Papiz et al. 2003). The central Mg atom in the monomeric Bchl *a* in the LH2 complex from *Phs. molischianum* is ligated to α-Aspartate-6, present in an amphiphilic N-terminal 3_10_ helix located at the membrane–water interface (Koepke et al. 1996). In both these LH2 complexes, the fact that this Mg-ligating bond (there are additional, less important stabilising contacts to these monomeric Bchl *a* molecules but these are omitted for brevity) originates from the end of the N-terminus results in the bacteriochlorin ring of the Bchl *a* being held in an orientation that is approximately in the plane of the membrane. In fact, the bacteriochlorin rings of the monomeric Bchl population from these two LH2s are tilted with respect to each other by about 20° and rotated by 90°. This does not happen in the *Rof. castenholzii* RC–LH complex. The Mg atom in the monomeric Bchl *a* is ‘conventionally’ liganded to the Histidine-26 residue in the β-helix and so this ensures that the bacteriochlorin ring adopts an orientation that is approximately normal to the membrane plane. The N-terminus of either polypeptide has no role in the binding of the monomeric Bchl in this complex (Fig. 1d).

This evolutionary distant member of the Chloroflexi produces a RC–LH complex with a LH ring that is a subtle hybrid of LH1 and LH2. However, there are still many open questions with regard to this complex; what is the genetic origin and sequence of helix X? How is the *puf*LM gene product processed so that it gives rise to three and not two proteins? Or is TM7 a completely independent protein that is coded for by its own gene? What is the correct status of the ‘missing’ carotenoid in the RC? These are just some of the uncertainties that need to be answered by further research on this interesting complex. Indeed, as *Roseiflexus* is a genus of bacteria in the family Roseiflexaceae, with *Rof. castenholzii* as the only known species. It is fascinating to consider what other species may be present in Nature, as yet undiscovered, and how they might have evolved.

### The *Gemmatimonas phototrophica* ‘Core’ structure

*Gemmatimonas *(*Gem.*)* phototrophica* is the only phototrophic representative as yet described of the bacterial phylum Gemmatimonadetes (Zeng et al. 2014). This species is an aerobic anoxygenic phototroph (AAP) meaning that it obtains its metabolic energy through respiration and only uses photosynthesis to supplement growth. These authors suggested that *Gem. phototrophica* gained this ability by a lateral gene transfer event of the ‘purple’ bacterial photosynthetic gene cluster. A high-resolution structure is not yet available; however, it is now known that this species has a remarkable RC–LH1 ‘Core’ complex that is much larger than the other single-RC complexes mentioned above. The RC–LH1 ‘Core’ from *Gem. phototrophica* contains approximately 62.4 ± 4.7 Bchl *a* molecules per RC and has a circular particle size diameter of approximately 190 Å and a molecular mass of around 800 ± 100 kDa (Dachev et al. 2017. By contrast, the LH1 ring in *Tch. tepidum* contains 32 Bchl *a* (2 per αβ-dimer) and has an elliptical size of 105 × 96 Å and a mass of some 426 kDa. The additional Bchl *a* molecules in the *Gem. phototrophica* RC–LH1 ‘Core’ are located in a second, outer concentric ring around a more ‘typical’ inner LH1 ring. The absorption spectrum of this double ring structure has Bchl *a* NIR Q_y_ absorption bands at 816 and 868 nm. It has been proposed that the 816 nm band arises from the outer ring and the 868 nm band comes from the inner ring. This then sets up an intra-molecular, downhill energy gradient in which light energy can be funnelled from the outer to the inner ring and on to the RC. In a sense, this complex makes a nice complement to the *Rof. castenholzii* RC–LH mentioned in the previous section. The *Rof. castenholzii* complex, functionally, has elements of an LH2 complex by incorporating the extra, weakly coupled Bchl *a* in the LH1 ring to produce an additional, blue-shifted absorption band. *Gem. phototrophica* goes one step further by producing an extra ring in its RC–LH1 ‘Core’, rather than making a separate LH2 complex. At present there is only a low-resolution, single particle image of this complex (Dachev et al. 2017). A higher resolution cryo-EM structure is eagerly awaited when, hopefully, a full structural description of the two populations of Bchl *a* molecules will allow their spectroscopic properties to be studied and understood.

## Final remarks

There are now a number of structures of RC–LH1 ‘Core’ complexes from different species of anoxygenic phototrophic bacteria. Remarkably, despite the evolutionary diversity involved, the differences highlighted above are elegant variations on a basic theme. In each case, the Bchl *a* molecules present in LH1 form a strongly exciton-coupled ‘ring(s)’ around the RC in a way that the distance to the Bchl *a* ‘special pair’ in the RC is short enough to allow fast and efficient energy transfer but long enough to completely prohibit electron transfer (Moser et al. 2003). Similarly, a single ‘gap’ in the LH1 ring would suffice to enable quinone/quinol exchange to take place. Yet, already, within this extremely small total number of structures available, wonderful and elegant solutions have evolved to connect the Q_B_ site with the bulk quinone pool in the membrane. Understandably, perhaps, previous work has concentrated on species that are/were classed as ‘purple’ bacteria. However, the novel structure of the RC–LH from *Rof. castenholzii* (Xin et al. 2018) and, particularly the concentric double ringed RC–LH1 complex from *Gem. phototrophica* (Dachev et al. 2017), suggests that further interesting complexes and adaptations remain to be discovered. Further work by biochemists, spectroscopists, theoreticians and physiologists is now needed to fully understand the benefits that these structural variations provide the different bacterial species in their specific ecological niches.

## Data Availability

Figures were prepared using legally licenced versions of Pymol or Origin 9.

## Abbreviations

AAP: Aerobic anoxygenic phototroph
AFM: Atomic force microscopy;
Bchl: Bacteriochlorophyll
*Blc*: *Blastochloris*
Bphe: Bacteriopheophytin
*Gem*: *Gemmatimonas*
ICM: Intra-cytoplasmic membrane
LH: Light harvesting
MQ: Menaquinone
NIR: Near infrared
PSU: Photosynthetic unit
*Phs*: *Phaeospirillum*
RC: Reaction centre
*Rbl*: *Rhodoblastus*
*Rof*: *Roseiflexus*
*Rsp*: *Rhodospirillum*
*Rps*: *Rhodopseudomonas*
*Tch*: *Thermochromatium*
UQ: Ubiquinone
